# Trabecular architecture in the forelimb epiphyses of extant xenarthrans (Mammalia)

**DOI:** 10.1186/s12983-017-0241-x

**Published:** 2017-11-29

**Authors:** Eli Amson, Patrick Arnold, Anneke H. van Heteren, Aurore Canoville, John A. Nyakatura

**Affiliations:** 1 AG Morphologie und Formengeschichte, Institut für Biologie, Humboldt Universität zu Berlin, Philippstraße 13, 10115 Berlin, Germany; 2 Bild Wissen Gestaltung. Ein Interdisziplinäres Labor, Humboldt Universität zu Berlin, Sophienstraße 22a, 10178 Berlin, Germany; 3 Institut für Spezielle Zoologie und Evolutionsbiologie mit Phyletischem Museum, Friedrich-Schiller-Universität Jena, Erbertstraße 1, 07743 Jena, Germany; 4 Sektion Mammalogie, Zoologische Staatssammlung München, Staatliche Naturwissenschaftliche Sammlungen Bayerns, Münchhausenstraße 21, 81247 Munich, Germany; 5 Steinmann Institute for Geology, Mineralogy, and Paleontology, University of Bonn, Nußallee 8, D-53113 Bonn, Germany

**Keywords:** Anisotropy, Bone, Epiphysis, Forelimb, Fossoriality, Functional adaptation, Mammals, Primates, Trabecular architecture, Xenarthra

## Abstract

**Background:**

Bone structure has a crucial role in the functional adaptations that allow vertebrates to conduct their diverse lifestyles. Much has been documented regarding the diaphyseal structure of long bones of tetrapods. However, the architecture of trabecular bone, which is for instance found within the epiphyses of long bones, and which has been shown experimentally to be extremely plastic, has received little attention in the context of lifestyle adaptations (virtually only in primates). We therefore investigated the forelimb epiphyses of extant xenarthrans, the placental mammals including the sloths, anteaters, and armadillos. They are characterised by several lifestyles and degrees of fossoriality involving distinct uses of their forelimb. We used micro computed tomography data to acquire 3D trabecular parameters at regions of interest (ROIs) for all extant genera of xenarthrans (with replicates). Traditional, spherical, and phylogenetically informed statistics (including the consideration of size effects) were used to characterise the functional signal of these parameters.

**Results:**

Several trabecular parameters yielded functional distinctions. The main direction of the trabeculae distinguished lifestyle categories for one ROI (the radial trochlea). Among the other trabecular parameters, it is the degree of anisotropy (i.e., a preferential alignment of the trabeculae) that yielded the clearest functional signal. For all ROIs, the armadillos, which represent the fully terrestrial and fossorial category, were found as characterised by a greater degree of anisotropy (i.e., more aligned trabeculae). Furthermore, the trabeculae of the humeral head of the most fossorial armadillos were also found to be more anisotropic than in the less fossorial species.

**Conclusions:**

Most parameters were marked by an important intraspecific variability and by a size effect, which could, at least partly, be masking the functional signal. But for some parameters, the degree of anisotropy in particular, a clear functional distinction was recovered. Along with data on primates, our findings suggest that a trabecular architecture characterised by a greater degree of anisotropy is to be expected in species in which the relevant epiphyses withstand a restricted range of load directions. Trabecular architecture therefore is a promising research avenue for the reconstruction of lifestyles in extinct or cryptic species.

**Electronic supplementary material:**

The online version of this article (10.1186/s12983-017-0241-x) contains supplementary material, which is available to authorized users.

## Background

Functional adaptations of bone structure reflect the lifestyle of vertebrates. Among tetrapods, the structure of long bones’ midshaft was primarily studied, and clear patterns, related to the aquatic or aerial environments for instance, were recognised (e.g., [1–4]). In comparison, and in the context of lifestyle adaptations, trabecular architecture has received little attention. Trabeculae are bony struts forming a lattice-like structure within skeletal elements. Also called spongy bone or cancellous bone, trabeculae are commonly found at the articular ends of long bones (epiphyses), where they form the core of the skeletal element [5]. It was shown experimentally that trabecular bone adjusts accurately and sensitively throughout life to the loads applied to the bone, as part of the ‘bone functional adaptation’, commonly referred to as ‘Wolff’s law’ (e.g., [6]; for a review see [7]). Trabecular parameters such as the number of trabeculae, their mean thickness, or their main direction of orientation (i.e., their anisotropy), hence have the potential to be highly insightful regarding the functional adaptations of a particular skeletal element.

Comprising diverse archosaurs (mostly birds) and mammals, the analysis of three-dimensional (3D) trabecular architecture with the largest taxonomic sampling was performed by Doube et al. [8], which was dedicated to the study of allometry (for a precursor study, see [9]; for a two-dimensional analysis, see [10]). The study of early ontogenetic stages in various taxa (horses and cow, [11]; dog, [12]; human, [13]) has provided insightful elements regarding the development of bone structure in relation to their different life histories. Experimental analyses used non-primate taxa (guinea fowl, [14]; potoroo, [15]; sheep, [6, 16]; mouse, [17, 18]; rabbit, [19]; dog, [20]) in order to test assumptions regarding bone functional adaptation. Almost all comparative functional analyses of 3D trabecular structure, however, were investigated in primates, which allowed compelling palaeoanthropological inferences, related for instance to bipedality [21] or tool use [22]. An exception focuses on horses and extinct relatives [23] but is mostly descriptive and did not analyse the 3D structure of the trabeculae. Chirchir [24] did include two carnivoran species in the dataset, but only investigated trabecular mass. Most recently, Mielke et al. (under review) investigated the 3D trabecular architecture in the femoral head of sciuromorphs (squirrels and close relatives), and did find significant differences among the lifestyle categories recognised therein. Extending our knowledge about non-primate taxa will be necessary to reach a broader understanding of trabecular architecture mechanical properties and function. The forelimb of xenarthrans offers a particularly appealing framework for that endeavour, as it comprises clear-cut differences in its functional use.

The most common approach to study 3D trabecular architecture is to define a region of interest (ROI) and describe quantitatively the trabecular bone that it comprises using various parameters (e.g., [25]; but see alternative whole epiphysis/bone approach [26] or ‘moving cube method’ [27]). Although not commonly acquired, one of these parameters is the main direction of anisotropy, which corresponds to the main orientation of the trabeculae (e.g., [28]).The latter is a fairly good proxy for the principal compressive strain (or principal load), at least in a cantilever-like loaded bone (e.g., calcanei of potoroo [15]; carpal/tarsal joints of sheep [6]; knee joint of the guinea fowl [14]). But counter-examples exist for bones loaded in a more complex way [29], and extreme positions (e.g., squatting in humans [30, 31]) might have a preponderant influence. Nevertheless, the main orientation of the trabeculae having furthermore successfully discriminated between primate locomotor types [32], we can assume that the comparison of the main direction of trabecular anisotropy among taxa having various uses of their limbs likely is of great relevance. The quantitative analysis of 3D trabecular architecture in general, and the direction of trabecular anisotropy in particular, has to date been conducted in relatively few taxa, but offered valuable insight into the functional significance of trabecular morphology. We therefore consider it as a promising avenue for research focused on comparative and evolutionary aspects of vertebrate morphology.

**Fig. 1:**
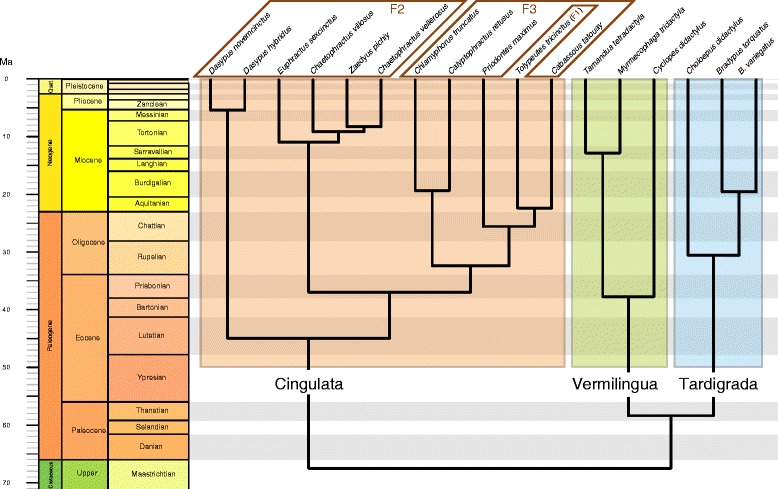
Timetree including the species herein sampled (data from Gibb et al. [77]). The lifestyle categories (fully arboreal sloths, intermediate anteaters, and fully terrestrial and fossorial armadillos) and fossorial categories (F1, F2, and F3 for least, intermediate, and highly fossorial, respectively) are represented with coloured polygons

Not only the xenarthrans are viewed as representing one of the four primary placental clades [33], but their lifestyles are also outstanding, involving classical examples of functional adaptations. Some of the most prominent of these, the focus of this work, are those that concern the forelimb. Indeed, each of the main extant xenarthran clades, namely sloths (Tardigrada), anteaters (Vermilingua), and armadillos (Cingulata) (Fig. 1), features a highly distinct forelimb use. Armadillos represent a textbook example of scratch-digging adaptation (e.g., [34]). Their digging skills were previously classified into three categories [35, 36], which can be abbreviated as ‘least fossorial (mainly cursorial)’ (category 1), ‘often dig, but digging not essential to their alimentation’ (category 2), and ‘burrowers and ant or termite eaters’ (category 3). However, it was recently documented that *Tolypeutes* (three-banded armadillo), which was classified as the least fossorial (category 1), is actually a burrower [37], emphasizing that all extant armadillos should be considered as efficient diggers. Armadillos’ forelimb posture is, to our knowledge, not well documented, but they are often regarded as unguligrade (*Tolypeutes*, *Priodontes*, the giant armadillo; see [38]) to sub-unguligrade (sensu [39]) for *Dasypus* (long-nosed armadillo) for instance (see tracks in [40]).

Anteaters practice a unique digging style, the so-called hook-and-pull [34, 41], well documented in *Myrmecophaga* (giant anteater) and *Tamandua* (lesser anteater; [34, 41]). *Cyclopes* (the silky anteater) seems to perform, at least partly, an analogous motion, as it uses its forelimb, especially its large claw, to pierce branches (Montgomery (1983) in [42]). *Myrmecophaga* sports a unique forelimb posture involving a vertical position of the manus and the transfer of the ground reaction force by flexed phalanges, approaching the ‘knuckle-walking’ practiced in chimpanzees and gorillas [43]. In contrast, *Tamandua* and *Cyclopes* both use another unique posture, the inverted-hand, during which the weight is essentially borne by the ulnar side of the hand (personal observations and [38] for *Tamandua*; [43] for *Cyclopes*). All anteaters are capable climbers (even *Myrmecophaga*, [44]). Although never quantified, we here view the anteaters’ forelimb as of intermediate mobility, i.e., involved in a greater range of movements than the armadillos, but not reaching the extreme mobility of sloths.

Extant sloths, or “tree sloths” comprise two genera, *Bradypus* (three-toed sloth) and *Choloepus* (two-toed sloth). The latter is assumed to be more closely related to the “ground sloths”; [45, 46]), so their numerous adaptations to a fully arboreal lifestyle and suspensory posture are assumed to be convergently acquired [47–49]. While there are some differences in the locomotion of the two genera of sloths ([48, 50], for further references see [51]), we will assume that their common suspensory posture involves similar movements and constraints, i.e., a highly mobile limb mostly loaded in tension with various loading directions.

These basic differences among the main clades of xenarthrans allow us to hypothesise clear differences in the loads withstood by their forelimb. In the sloths’ forelimb, we assume that the loads are of more diverse directions than those of armadillos, and, in turn, that in armadillos a main loading direction might be present (clearly greater than secondary loadings of different directions). We expect for the anteaters to be intermediate in that regard. The strenuous burrowing habits of armadillos have been suggested to be reflected in several peculiarities of their postcranium including but not limited to the presence of xenarthrales [52]. We therefore hypothesize a distinct main loading direction (involving lesser importance for the other loading directions) to occur in the forelimbs of armadillos. Nevertheless, it is noteworthy that anteaters, especially *Myrmecophaga*, are capable of striking extremely strong blows with the forelimb [53]. It is in this framework that we will interpret the differences (if any) among the trabecular parameters within regions of interest of the main epiphyses of the forelimb of armadillos, anteaters, and sloths.

## Methods

### Specimens and functional categories

Only skeletally mature individuals, i.e., in which the epiphyseal (growth) plate of the studied epiphysis is resorbed (except in the distal radius of armadillos where a remnant of internal epiphyseal line was observed as persisting through adulthood in some species) were sampled from Museum collections (see list of abbreviations). In addition, specimens with apparent bone diseases or from zoos (as indicated by the specimen labels) were not sampled (only one specimen probably came from a zoo, *Euphractus sexcinctus* ZMB_85883, but for all parameters it fell either within the specific range or very close to it), as it may influence bone structure. Both right and left limbs were sampled, as handedness (if any) was unknown (but see its possible effect on trabecular parameters in [54]). Similarly, both sexes were sampled indifferently. All extant genera of xenarthrans are present in the final dataset, and replicates were acquired for most sampled species, representing in total (after exclusion of immature specimens) 17 species and 43 specimens (Fig. 1, Additional file 1). We sampled the scapula, humerus, and radius. The hand was not included in the analysis because in the elements of the smaller taxa too few trabeculae were observed, involving the exclusion of an important proportion of the dataset.

We defined two schemes of functional categorisations (Fig. 1). The first involves one category per main lifestyle, the fully arboreal sloths, intermediate anteaters, and fully terrestrial and fossorial armadillos. The second scheme, which only concerns the armadillos, involves three previously defined fossorial categories (see above and [35, 36]): category 1, the least fossorial three-banded armadillo *Tolypeutes* (but see [37]), category 2, an intermediate category comprising the Dasypodidae and Euphractinae, and category 3, the highly fossorial armadillos comprising the Chlamyphorinae and Tolypeutinae except *Tolypeutes*. Both lifestyle and fossorial categories will be commonly referred to as functional categories. As defined here, these categories are either perfectly (lifestyle categories) or almost perfectly (fossorial categories) matching phylogeny, i.e., neither lifestyles nor fossoriality are represented by several convergent acquisitions (except in the two genera of sloths where an arboreal lifestyle was most likely acquired independently). This results in the fact that, strictly speaking, one cannot separate a functional feature of one of the categories from a phylogenetic attribute (in other words, functional and phylogenetic effects cannot be distinguished). However, given the extreme sensitivity and plasticity of the trabecular bone (e.g., [6, 55]), and given that Doube et al. [8] did find that among their large dataset of amniotes the phylogeny bore only minor influence on trabecular parameters, we expect that the phylogenetic relationships among xenarthrans will not conspicuously influence their trabecular architecture. Obtaining a significant phylogenetic signal (as defined by Blomberg et al. [56]) and observing a pattern in the traits’ distribution that cannot obviously be attributed to functional differences (i.e., within a functional category closely related species would be more similar to each other than to other species) would invalidate this hypothesis. That is why we tested for the presence of a phylogenetic signal within subsamples consisting of individual functional categories (see below).

### Data acquisition

All specimens were scanned using micro computed tomography (μCT) [Tomoscope Synergy Twin, Experimental Radiology Lab, Institute of General and Interventional Radiology, Jena University Hospital; phoenix|X-ray Nanotom m, Zoologische Staatssammlung München; phoenix∣X-ray v∣tome∣x s 240, Steinmann-Institut, Bonn; all Germany], with a resolution of 17–97 μm (mostly depending on the size of the object; see resolutions in Additional file 1). Differences in the resolution can influence the calculation of the trabecular parameters ([57] and references therein), hence our assessment of the relative resolution, as explained below. In each case, the quality of the scans (resolution and contrast) was checked visually before and after the ROI extraction and thresholding (see below). Greyscale 16-bits stacks were hence obtained and processed with the Fiji package [58], a combination of ImageJ (in this case ImageJ2 v. 1.51 g) and plugins [59, 60]. Quality assessment of the scans was done after the acquisition of the trabecular parameters (because two of them are necessary to do so, the Connectivity and trabecular mean thickness (Tb.Th), see below). The number of specimens eventually analysed (after this quality assessment) for some taxa is rather low (Table 1), with a mean number of specimens per genus of 3.1, which can be viewed as a limitation of the present study.

**Table 1:** Number of specimens included in the analysis (after quality assessment of the scans, see Methods section)

	Genera	Species	N
Cingulata			25
	*Cabassous*		1
		*Cabassous tatouay*	1
	*Calyptophractus*		2
		*Calyptophractus retusus*	2
	*Chaetophractus*		5
		*Chaetophractus vellerosus*	3
		*Chaetophractus villosus*	2
	*Chlamyphorus*		3
		*Chlamyphorus truncatus*	3
	*Dasypus*		2
		*Dasypus hybridus*	1
		*Dasypus novemcinctus*	1
	*Euphractus*		3
		*Euphractus sexcinctus*	3
	*Priodontes*		5
		*Priodontes maximus*	5
	*Tolypeutes*		2
		*Tolypeutes tricinctus*	1
		*Tolypeutes* sp.	1
	*Zaedyus*		2
		*Zaedyus pichiy*	2
Vermilingua			12
	*Cyclopes*		3
		*Cyclopes didactylus*	3
	*Myrmecophaga*		5
		*Myrmecophaga tridactyla*	5
	*Tamandua*		4
		*Tamandua tetradactyla*	4
Tardigrada			6
	*Bradypus*		4
		*Bradypus variegatus*	2
		*Bradypus torquatus*	1
		*Bradypus* sp.	1
	*Choloepus*		2
		*Choloepus didactylus*	2
Total			43

Successive ‘Re-slicing’ routines and in some cases image flip were used to place all bones in the same standard orientation: mediolateral in the X-axis, medial towards the left of the image; anteroposterior in the Y-axis, anterior towards the top of the image; and proximodistal in the Z-axis, proximal towards the top of the stack. The scapula was oriented so that the greater length of glenoid cavity (from the side bordered by the postscapular fossa to the side of the coracoid process) would be aligned on the Y-axis (anterior towards the top of the image), and lesser length (from subscapular border to the scapular spine border) would be aligned on the X-axis (medial towards left; see orientation of 3D model in the Additional file 2). For the humerus, the head was set to face downward (maximal curvature of the head is normal to the Y-axis), and the centres of the proximal and distal metaphyses were aligned on the Z-axis (proximal towards the top of the stack; see Additional file 3). For the radius, the greatest mediolateral length of the distal trochlea was aligned on the X-axis, posterior side facing downward, and, as for the humerus, the centres of the proximal and distal metaphyses were aligned on the Z-axis (see Additional file 4).

**Fig. 2:**
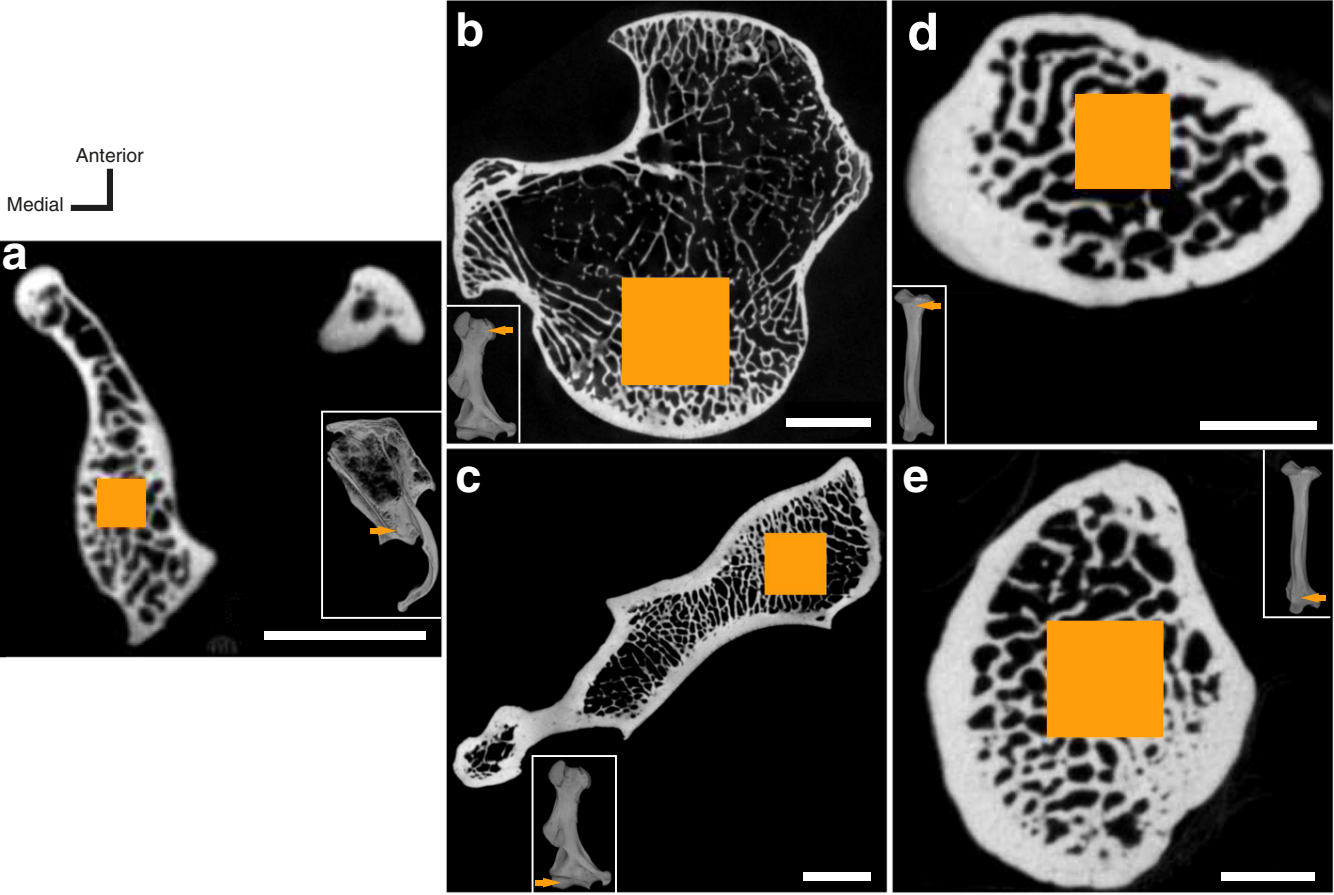
Selection of the regions of interest (ROIs). Transverse virtual sections (CT-scans in the X-Y plane) of the studied bones oriented as for the data acquisition, where the area coloured in orange indicates the central (transverse) slice in the ROI. **a** glenoid fossa (scapula of *Chlamyphorus truncatus* ZMB_Mam_6007), scale bar = 3 mm; (**b**) and (**c**) humeral head and capitulum, respectively (*Cabassous tatouay* SMNS-26661), scale bars = 5 mm; (**d**) and (**e**) radial head and trochlea, respectively (*Euphractus sexcinctus* SMNS-26660), scale bars = 2 mm. Three-dimensional (3D) renderings of the relevant bones (right bones seen in lateral view for the scapula and anterior view for the humerus and radius) are displayed as insets. The anatomical orientations (‘Anterior’, ‘Medial’) are only valid for the sections (not for the 3D renderings). ROIs were selected to be as large as possible but excluding the cortex. Note that the slices displayed (centre of each ROI) do not appear as comprising the maximal quantity of trabeculae because the ROI selection was always restricted at its proximal or distal end. See also 3D models in Additional files 2, 3, 4

The rest of the procedure was performed with the BoneJ plugin [61]. Cubic ROIs were selected with the ‘Fit Sphere’ routine, so that the largest cube included in the sphere (referred to as ‘Inner Cube’ in the plugin) would be as large as possible but without including cortical bone. This acquisition of the ‘bulk’ of the trabeculae in the epiphyses was favoured over other methods involving a constant or scaled ROI volume (e.g., [25]), in order to sample as much trabeculae as possible, which was necessary for the epiphyses of small-sized xenarthrans that comprise a very limited number of trabeculae. Therefore, in order to sample functionally analogous regions in all species, this was applied across the whole dataset. For the glenoid cavity (scapula), this ROI was placed just proximal to the articular surface, and mediolaterally and anteroposteriorly centred relative to the cavity (Fig. 2a; Additional file 2). For the humerus, the proximal and distal ROIs were respectively centred in the head and capitulum (Fig. 2b and c, respectively; Additional file 3). The proximal ROI of the radius was placed just distal to the proximal articular surface, and mediolaterally and anteroposteriorly centred relative to the head (Fig. 2d; Additional file 4). The distal ROI of the radius was placed just proximal to middle of the trochlea (the latter being strongly inclined in some taxa; Fig. 2e; Additional file 4).

The extracted ROI (a cubic substack) was thresholded with the ‘Optimise Threshold > Threshold Only’ routine. After purification of the substack (‘Purify’ routine), the corresponding routines of BoneJ were then used to measure ten trabecular parameters, namely the degree of anisotropy (DA), main direction of the trabeculae (herein called MDT; see also Mielke et al. (under review)), the Connectivity (only used for the scan quality assessment) and connectivity density (Conn.D), bone volume (BV), total volume of the ROI (TV), trabecular mean thickness (Tb.Th), trabecular mean spacing (Tb.Sp), bone surface area (BS), and average branch length (Av.Br.Len).

**Fig. 3:**
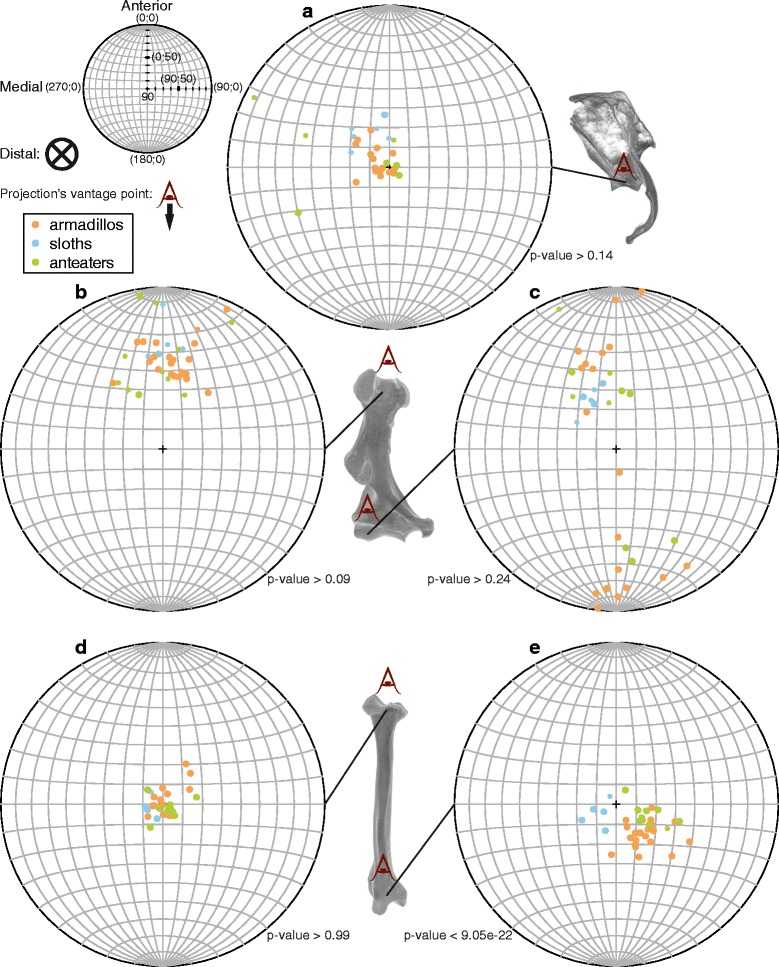
Main direction of the trabeculae (MDT) in regions of interest (ROIs) located in the epiphyses of the forelimb of xenarthrans. Stereographic projections of the MDT colour-coded according to the lifestyle categories for each ROI, namely the glenoid cavity (**a**), humeral head (**b**), humeral capitulum (**c**), radial head (**d**), and radial trochlea (**e**). The projections are on the lower hemisphere, which corresponds to the distal direction (symbolised on the upper left legend by a crossed circle). The smaller dots represent specimens for which the degree of anisotropy (DA) is below 0.5, greater values being represented by the larger dots. Three-dimensional (3D) renderings of the relevant bones (right bones seen in lateral view for the scapula and anterior view for the humerus and radius) indicate the location of the ROIs and the perspective of the projections (‘eye symbol’). The anatomical orientations (‘Anterior’, ‘Medial’, and ‘Distal’) are only valid for the stereographic projections (not for the 3D renderings). On the upper left smaller projection (used to indicate the anatomical direction) are labelled the pairs of coordinates that define the 3D vectors (azimuth; plunge), except for the centre (perfect distal orientation) for which the azimuth is irrelevant. The *p*-values indicate the significance of the difference among functional categories (ANOVA for spherical data)

DA (no units) involves the acquisition of eigenvectors and eigenvalues, which define the ellipsoid with which the main, intermediate, and least orientation of the trabecular anisotropy are represented (as defined by the mean intercept length method [62]); DA = 1–1/(ε_1_/ε_3_), with ε_1_ and ε_3_ the greater and lesser eigenvalues, so if no preferential alignment of the trabeculae is present DA = 0 and if perfect alignment is present DA = 1; in other studies, ε_1_/ε_3_ is used directly (see ‘Discussion’ section). The MDT was associated with the 3D vector of the main direction of orientation of the trabecular anisotropy (given by the first eigenvector, which is the first column of the matrix outputted by the ‘Anisotropy’ routine). An azimuth (or trend) and plunge (or inclination) was deduced from the x, y, z eigenvector components using a custom R function (Additional file 5). This way, the MDT can be represented in a stereographic projection (see [28]; Fig. 3, using the RFOC package [63], see below), in which a dot represents a vector departing from the centre of a sphere and projected, as oriented here, on its lower hemisphere (positive on the Z-axis). According to our orientation, the centre of the projection denotes the distal direction (periphery is hence representing vectors perpendicular to the proximodistal axis), its right side (positive on X-axis) denotes the lateral direction, and its upper side (positive on Y-axis) denotes the anterior direction.

The Connectivity (discrete number) approximates the number of trabeculae, and Conn.D is the connectivity per unit of volume (in mm^−3^). BV, in mm^3^, is the volume of the ROI occupied by bone. TV being also in mm^3^, BV/TV has no units. The Tb.Th and Tb.Sp are both in mm. BS is in mm^2^, so its ratio to TV, BS/TV, is in mm^−1^. Once the above-mentioned parameters were acquired, the stack was skeletonised (with the ‘Skeletonise 3D’ routine) in order to measure an additional parameter, the Av.Br.Len, which is in mm. All raw measurements are given in the Additional file 1.

To assess the quality of the scans, we used the relative resolution defined by Sode et al. [64] as the number of pixels representing the average thickness of the trabeculae (Tb.Th divided by scan resolution). The average relative resolution for the whole dataset is 8.1, the values spanning from 4.2 to 62.2. As recommended by Kivell et al. [57], these values roughly equal or exceed clinical high-resolution scans, and were hence considered as appropriate. The parameters of some small-sized taxa were clearly outlying, because of the small number of trabeculae included in the corresponding ROI (even though the largest possible ROI was selected, see above). Therefore, we used the values of the Connectivity parameter (which approximates the number of trabeculae) to set a threshold for each ROI below which the specimens were excluded.

Three-dimensional models (Additional files 2, 3 and 4) were produced with the 3D viewer plugin [65] of Fiji.

### Statistical analyses

The analyses involve traditional (non-phylogenetic), spherical, and phylogenetically informed statistics. All computations were performed with R v. 3.4 [66]. Significance threshold was set at 5% (Holm–Bonferroni method of correction for multiple testing was applied when warranted).

#### Traditional statistics

**Fig. 4:**
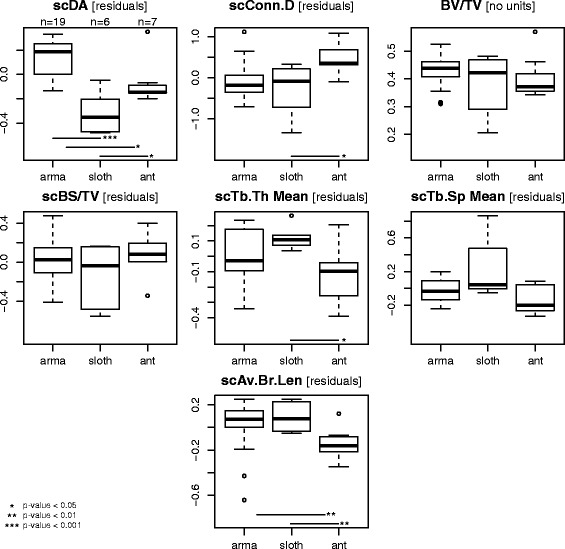
Distribution of the non-directional trabecular parameters of the glenoid cavity among the xenarthran lifestyle categories. If the parameter was size-corrected, “sc” precedes its abbreviation, and it is the residuals of the regression of the original parameter against a body size proxy (TV) that are used and plotted (see original parameters’ units in the text). Note that a phylogenetic ANCOVA was warranted in the case of the scDA. Abbreviations: arma, armadillos; sloth, sloths; ant, anteaters. Sample size is only given for scDA but is valid for the other parameters as well. Here all specimens are included (outliers represented by small circles), except for those that were initially excluded based on the small number of trabeculae (see ‘Data acquisition’ section)

Pairwise comparisons among the functional categories were performed, when relevant, with the size-corrected trabecular parameters. TV was used as a body size proxy because it is directly measured on the specimens (contrary to body mass specific mean) and because it essentially scales isometrically to body mass in our dataset: Slopes of ordinary least squares regressions (lm function) of TV against body mass (using specific means from [67–70]) for each ROI are all different from 0 (*p*-values <1e-07) and not significantly different from 1 (using the Student t distribution, pt function, *p*-values >0.24), except for the radial head for which the *p*-value >0.045. Size-correction was performed using unpooled ordinary least squares regression ((Reduced) Major Axis regression was shown to be biased in such a case [71]) of each parameter against the body size proxy (TV), with both variables log-transformed. When the slope was significantly different from 0 (i.e., there is a significant correlation with size), the residuals of the regression were recovered and considered to represent the ‘size corrected’ parameter (e.g., [72]; see which parameters were concerned for each ROI in Fig. 4 and Additional file 6). If the studied parameter of the functional categories was normally distributed (shapiro.test function) and the variances were homogenous (bartlett.test function), a traditional analysis of variance (ANOVA) (aov function) and Tukey’s post hoc test (TukeyHSD function) were used. If the variances were not found as homogeneous, several t-tests were used [for each pair of category, a Levene’s test (leveneTest function, car package [73]) was used to determine if a Welch’s t-test should be used], and the *p*-values were corrected for multiple testing (p.adjust function). Departure from normality was sometimes found and caused by outliers. In those cases, the latter were excluded (outlier function, outliers package [74]). Boxplots were produced with the boxplot2 function (gplots package [75]).

#### Spherical statistics

The main direction of the trabeculae, MDT, was visualised with stereographic projections (net and focpoint functions, RFOC package [63]). The vectors (as defined by the x, y, z eigenvector components) were normalised (i.e., made unit vectors), and if necessary, inversed (for all vectors to point distally, i.e., positive along the Z-axis). After that the equality of concentration was tested (spherconc.test function, same package), differences among functional categories were assessed with an ANOVA for spherical data (Directional package, hcf.aov function [76]).

#### Phylogenetically informed statistics

If warranted (see phylogenetic signal below), pairwise comparisons were performed within a phylogenetically informed framework. For terminal taxa (timetree tips, species in our case) represented by multiple specimens, the mean of each parameter was used in the subsequent operations. Two specimens that were identified only up to the generic level were excluded. We used the timetree of Gibb et al. [77], pruning the unstudied species (read.nexus and drop.tip functions, ape package [78]). Matching between the phylogeny and the parameter data was checked with the name.check function (Geiger package [79]). A visualization of the timetree (Fig. 1) was performed with the geoscalePhylo function (strap package [80]). In order to decide whether phylogenetically informed tests were warranted or not, we computed Pagel’s lambda, a measure of phylogenetic signal (with the phylosig function of the phytools package [81]), using the residuals of a linear regression (lm function; see [82]) of, for each ROI, each parameter against the body size proxy TV (both log-transformed). The choice of Pagel’s lambda, of which a value of 1 denotes that the trait under study evolved as expected under a Brownian motion model, was motivated by the fact that the subsequent analyses will use its value to phylogenetically inform the tests (see below). When a significant phylogenetic signal was detected, these residuals were mapped on the phylogeny (contMap function, phytools package [81]) to visualise the evolutionary pattern of each trait. To test whether or not the parameters differ among the functional categories, we performed phylogenetically informed analysis of covariance (ANCOVA) (using generalised least squares linear models, gls function of the nlme package [83]), with the body size proxy as a covariate, and a within-group correlation structure based on the optimised lambda value (see [82]; with the corPagel function, ape package [78]), because it is assumed that a transformation of the topology according to this value makes the parameter’s evolution best fit the Brownian motion model [84]. The ‘maximum likelihood’ method was used, except when it was not able to recover the optimised lambda. In the latter case, it is the ‘restricted maximum likelihood’ that was used (for the present dataset the latter recovered reasonable values of lambda and the resulting gls models yielded the same results as the ‘maximum likelihood’ method when both methods could have been employed). The residuals of the gls fit were tested for normality (qqnorm) and homoscedasticity (plot function, to visualise the standardised residuals versus fitted values; see recommendation of [85]), and, when warranted, outliers were excluded and another fit was performed. For those parameter-ROI couples for which a significant phylogenetic signal was recovered, we measured again the phylogenetic signal (using size corrected values if warranted) but within two subsamples, one consisting of the largest functional category (i.e., fully terrestrial and fossorial lifestyle, the armadillos) and the other of the least inclusive most speciose category (i.e., intermediate fossorial category). This was performed in order to roughly assess whether phylogeny is a factor intrinsically affecting trabecular parameters. The phylogenetic signal was not investigated in the other functional categories because of their small number of terminal taxa that would have likely resulted in little power for the tests.

## Results

### Main direction of the trabeculae (MDT)

The MDT (as given by the main direction of trabecular anisotropy) differs rather consistently among the ROIs (Fig. 3). For the glenoid cavity of the majority of species (Fig. 3a), the MDT is mostly oriented proximodistally. The distinction among the lifestyle categories is poor. The sloths cluster with a slight anterolateral component in their MDT, but partly overlap with the distribution of armadillos. Three anteaters feature an outstandingly weaker distal component in their MDT. For the humeral head (Fig. 3b), the MDT is consistently distoanterior, with no clear lifestyle categories distinction. The humeral capitulum (distal epiphysis, Fig. 3c) is the ROI with the greatest variation in MDT, the directions being clustered in two zones, one distoanteromedial and another distoposterolateral. Beside the fact that sloths are only found in the former, there is no clear distinction among the lifestyle categories. The radial head’s ROI (Fig. 3d) is the one for which the MDTs are least disparate among xenarthrans, most specimens featuring a mostly proximodistal direction. Again, no clear distinction is found among the lifestyle categories. Finally, and contrary to previous ROIs, the radial trochlea (Fig. 3e) discriminates lifestyle categories, as indicated by a spherical ANOVA (*p*-value <9.5e-22; other ROIs, *p*-values >0.09). While MDT in all specimens is again mostly proximodistally oriented, the sloths feature a slight medial component, the anteaters a slight lateral component, and the armadillos a slight posterolateral component. One should note that in some cases a conspicuous intraspecific variability is observed. No clear difference is found in any of the ROIs among the fossorial categories (not shown).

### Non-directional trabecular parameters

The DA, Conn.D, BV/TV, BS/TV, Tb.Th, Tb.Sp, and Av.Br.Len show a rather large variation among xenarthrans (Fig. 4; Table 2; Additional files 6 and 7). Most of these parameters for most of the ROIs were significantly correlated to body size (as demonstrated by the linear regressions against the body size proxy TV). Indeed, only the BV/TV of the glenoid cavity, humeral head and capitulum, and radial trochlea are not correlated to size (the radial head stands out among ROIs because BV/TV is affected by scaling, while Conn.D, Tb.Th and Tb.Sp are not). Size was hence taken into account (when relevant) for the following comparisons. A significant phylogenetic signal was found in only one parameter (DA), suggesting that the phylogenetic relationships among xenarthrans do not preponderantly affect their trabecular parameters.

**Table 2:** Mean xenarthran untransformed values of the non-directional trabecular parameters for each region of interest

	DA	Conn.D	BV/TV	BS/TV	Tb.Th mean	Tb.Sp mean	Av.Br.Len.
	(NU)	(nb/mm^3^)	(NU)	(mm^−1^)	(mm)	(mm)	(mm)
Glenoid							
All	0.68	18.47	0.42	3.96	0.22	0.38	0.34
Armadillos	0.81	22.84	0.43	4.51	0.20	0.32	0.32
Anteaters	0.53	14.52	0.40	3.32	0.24	0.40	0.34
Sloths	0.44	9.24	0.38	2.97	0.27	0.55	0.39
Humeral head							
All	0.52	11.68	0.43	3.36	0.26	0.46	0.38
Armadillos	0.60	12.35	0.41	3.38	0.25	0.47	0.38
Anteaters	0.40	11.59	0.45	3.43	0.26	0.41	0.37
Sloths	0.43	9.36	0.44	3.14	0.31	0.49	0.40
Humeral capitulum							
All	0.66	9.90	0.47	3.32	0.32	0.48	0.42
Armadillos	0.77	10.63	0.48	3.57	0.31	0.47	0.44
Anteaters	0.51	10.06	0.47	3.24	0.32	0.44	0.39
Sloths	0.54	7.18	0.42	2.65	0.34	0.58	0.42
Radial head							
All	0.77	7.95	0.47	3.57	0.28	0.42	0.42
Armadillos	0.87	5.71	0.50	3.84	0.30	0.41	0.45
Anteaters	0.74	11.25	0.45	3.53	0.26	0.38	0.38
Sloths	0.57	8.07	0.41	2.81	0.29	0.51	0.41
Radial trochlea							
All	0.72	13.82	0.46	3.50	0.33	0.41	0.40
Armadillos	0.79	16.05	0.49	3.87	0.34	0.37	0.39
Anteaters	0.63	11.19	0.44	3.20	0.30	0.44	0.42
Sloths	0.56	8.75	0.40	2.39	0.30	0.52	0.40

Footnotes: Abbreviations: *Av.Br.Len* average branch length, *BS* bone surface, *BV* bone volume, *Conn.D* connectivity density, *DA* degree of anisotropy, *Tb.Th* trabecular mean thickness, *Tb.Sp* trabecular mean spacing, *NU* no units

**Fig. 5:**
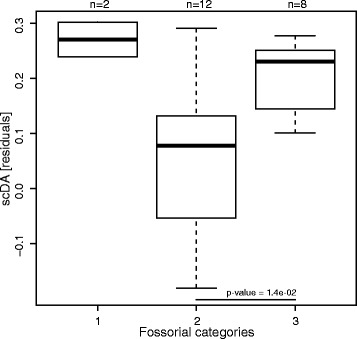
Differences in the size-corrected (using residuals of the regression of the original parameter against a body size proxy, TV) degree of anisotropy (scDA) of the humeral head among armadillo’s fossorial categories: 1, supposedly least fossorial (only *Tolypeutes*); 2, intermediate (Dasypodidae and Euphractinae); and 3, highly fossorial (Chlamyphorinae and Tolypeutinae except *Tolypeutes*). Only one pairwise comparison was performed and is indicated by the horizontal bar and *p*-value

DA (no units) shows a significant phylogenetic signal in all ROIs (*p*-values <0.03) except those of the radius (*p*-values >0.44). This parameter yielded a clear lifestyle distinction in all ROIs, which show the same pattern, namely that armadillos have a greater DA than non-armadillos (phylogenetic ANCOVA if warranted or size-corrected pairwise comparison; see boxplots in Fig. 4, Additional file 6; see all *p*-values in Additional file 8). Furthermore, for the radial head, the DA of anteaters is significantly greater than in sloths, describing a gradient from the most fossorial armadillos with greatest DA to the non-fossorial sloths with the lowest DA. Only in the humeral capitulum, the phylogenetic ANCOVA yielded a significant influence of size on DA. However, the two categories (armadillos/non-armadillos) did not differ in size (t-test on TV), so the difference in the response variable (DA) can be directly imputed to the explanatory variable (lifestyle). Among armadillos, the DA significantly differs among fossorial categories only for the humeral head. In the humeral head (Fig. 5), the highly fossorial armadillos (category 3) feature a significantly greater DA than the intermediate ones (category 2). Surprisingly, *Tolypeutes*, argued to be the least fossorial armadillo, feature one of the greatest DA of our sample. In the humeral capitulum and the radial head, the highly fossorial armadillos again feature a greater DA than those of the intermediate category, but the difference is not found as significant (in these cases *Tolypeutes* falls within the range of the intermediate category). Both subsamples investigated to test the overall influence of phylogeny in our dataset (see ‘Methods’ section) yielded a very low lambda and non-significant *p*-value of the test for the presence of a phylogenetic signal.

Conn.D values (mm^−3^; size-corrected except for the radial head) are essentially found as greater in anteaters than in the other xenarthrans. This is significant in the glenoid cavity (Fig. 4). In the humeral ROIs and the radial head (Additional file 6), only the comparison with armadillos is found as significant, but this is explained by the rather wide range of variation of sloths, which are essentially intermediate. There are no obvious differences among the lifestyle categories for the radial trochlea ROI. No differences were found among the fossorial categories for any of the ROIs either.

BV/TV (no units) poorly discriminates the functional categories (i.e., neither the lifestyle nor the fossorial categories; Fig. 4, Additional file 6). For the humeral ROIs, it is found as greater in armadillos than in sloths (anteaters fall roughly in between, but with a rather important range of variation), a comparison that is significant only for the humeral capitulum. No significant differences were found among the lifestyle categories for the other ROIs or among the fossorial categories for any of the ROIs. One can note that in the supposedly least fossorial *Tolypeutes*, the BV/TV values are particularly low.

BS/TV (mm^−1^) yields a functional discrimination but that is not consistent across the studied ROIs. Among lifestyle categories, it is only for the humeral head that armadillos have significantly lower values than the other xenarthrans (Additional file 6). Among the fossorial categories, and in the glenoid cavity, there is a clear gradient with the lowest values for the least fossorial armadillos to the highest values for the most fossorial ones (the difference between categories 1 and 2 is however not significant, with a *p*-value = 0.053).

The Tb.Th and Tb.Sp (both in mm) yield no clear discrimination among the lifestyle categories (Fig. 4, Additional file 7) or fossorial categories. This might be attributed to the strong size effect, consistently found except for the radial head ROI. One should note, however, that there is a tendency for the Tb.Th (normalised with the body size proxy TV) of armadillos to be greater than that of other xenarthrans.

Finally, the Av.Br.Len (mm) is essentially found as lower in anteaters than in the other lifestyle categories. This is significant for the glenoid cavity (Fig. 4) and humeral head, and only between the anteaters and armadillos for the humeral capitulum and radial head (Additional file 6). In the latter cases, the sloths are generally intermediate. Among the fossorial categories, it is only in the radial head that a significant difference is found, namely a greater Av.Br.Len in the highly fossorial armadillos.

## Discussion

The chief goal of the present study is to characterise the trabecular architecture of the main epiphyses of the forelimb of xenarthrans, and by that means reaching a better functional understanding of the trabecular parameters. Our expectation was for the influence of phylogenetic relationships to be marginally represented in the trabecular architecture of xenarthrans (or any other clade). Indeed, no significant phylogenetic signal was found in any of the parameters and for any ROIs, except for the DA. Again, one cannot strictly differentiate functional from phylogenetic signal in our case, but we interpret this result as a good indication that the functional signal is preponderant in the trabecular architecture, as DA yielded by far the clearest differentiation among the functional categories. One cannot exclude that only DA is intrinsically affected by phylogeny. However, we view this as unlikely, as closely related taxa within functional categories did not resemble each other more than less closely related taxa for this parameter (low lambda value and non-significant phylogenetic signal in the studied subsamples). Since we also accounted for size, which has an important (structural) effect on the trabecular architecture [8, 86], we hence consider that the differences we found among the functional categories (either lifestyle or fossorial categories) are most likely of functional significance.

As Doube et al. [8] found across mammals and birds, and as Ryan and Shaw [86] found in primates, an important scaling effect among the trabecular parameters was observed in xenarthrans. Furthermore, conspicuous intraspecific variability was observed in some cases, which could have masked the functional signal in those cases in which it was not recovered. This seems particularly obvious for the MDT, which discriminated lifestyle categories only in one ROI out of five (the radial trochlea).

Previous analyses of xenarthran bone structure tackled long bone mid-diaphysis (using 2D approaches [87–89]). They revealed that the midshaft of xenarthrans is characterized by a rather high global compactness (when compared to the generalized mammalian condition) and, in some taxa, by the presence of a spongiosa that fills the medullary cavity. On the whole, our observations on the trabecular architecture at the epiphyses did not reveal any major patterns that are departing from that of other mammals, i.e., epiphyses filled with trabecular bone that is surrounded by a rather thin cortex. In the following, relevant comparisons with other taxa for which 3D trabecular architecture was assessed (virtually only primates) are performed for each of the studied ROIs (no comparative data were found for the humeral capitulum).

### Glenoid cavity

The MDT in the glenoid cavity of humans was estimated but never with a method directly comparable to the one used herein. However, it was described that the trabeculae are oriented radially, perpendicular to the subchondral plate (aligned along the mediolateral axis for human scapula orientation; [90–92]). The mostly proximodistally oriented trabeculae (usually with a high DA) of xenarthrans (Fig. 3a), especially armadillos, might reflect, as opposed to that of humans, their quadrupedal posture.

### Humeral head

The humeral head (along with the humeral capitulum) was the ROI that yielded the best lifestyle discrimination. Several analyses also used a ROI representing the bulk of the trabeculae of the humeral head in primates [93–95]. In our analysis, DA is on the whole the only parameter that consistently discriminates lifestyle categories, and for some ROIs, fossorial categories. A similar conclusion was drawn by Fajardo and Müller [93], who investigated the humeral (and femoral) head in suspensory-climbing and more quadrupedal primates. One should note that their overall values of degree of anisotropy are lower than what we recovered (eigenvalues ratio ranges from 1.12 to 1.44, which corresponds to a DA range of 0.11–0.31), their most anisotropic specimen falling within the range of the sloths and anteaters (i.e., non-armadillo xenarthrans, which are less anisotropic than armadillos). In the orangutan, chimpanzee, and human [94] and in different human populations [95], relatively low DA values close to those found by Fajardo and Müller [93] (hence relatively low when compared to xenarthrans) were recovered. The anthropoid dataset of Ryan and Walker [96] is marked by slightly greater DA values (using the alternative method “SVD DA”, which corresponds to a range of 0.32–0.53 for the DA according to our use), which is probably due to their use of a smaller ROI (1/10th the volume of the best-fit articular surface sphere). A relatively low DA was also found in the dog ([97]; therein eigenvalues ratio = 1.30, so DA = 0.23), but one should note that the method used by these authors is quite different (e.g., the ROI was physically extracted, etc.). Regarding a relationship with lifestyle, the DA was found as higher in the more terrestrial primates by Fajardo and Müller [93]. In Scherf et al.’s [94] case the more terrestrial chimpanzee was the taxon characterised by the lowest value. While there were no significant differences between modern humans and the presumably more active Neolithic humans [95], the Neolithic females were found as having a greater DA, which was interpreted as indicative of their more specialised working routine. Ryan and Shaw [25] used a multivariate approach, and did not recover clear functional differences in the parameters of the humeral head of primates (as in [98]). However, the tendency was also for quadrupedal and terrestrial taxa (and some arboreal taxa) to have a more anisotropic trabecular architecture. All in all (except for the data of Scherf et al. [94]), there seems to be a positive correlation (or relationship) between DA and the presence of a main loading direction, which in turn might reflect a more restricted range of movements. This fits biomechanical expectations [99]. Based on the comparison among armadillos’ fossorial categories, we are further able to argue that an even greater DA would be associated with a more derived digging adaptation, which might involve an even more distinct main loading direction (i.e., magnitudes of other loading directions are of much lesser importance). One should note, however, that *Tolypeutes*, argued to be the least fossorial armadillo, featured among the greatest DA values for the humeral head ROI. The correlation just mentioned is, hence, either not valid in this case, or this taxon is more fossorial than previously thought, which has already been suggested by Attias et al. [37]. Although it concerned the femoral head, one should note that the DA in non-leaping (arboreal) primates was found to be lower than in the leaping ones, with the slender loris featuring the lowest mean value [32].

Conn.D was found to be greater in anteaters than in the other xenarthrans. This is not easy to interpret functionally, as anteaters’ lifestyle is considered herein as somewhat intermediate between that of armadillos and that of sloths. One can note that it is consistent that we also found Av.Br.Len to be lower in anteaters, as more trabeculae per unit of volume without significant change in their thickness or spacing implies that they are shorter. While to our knowledge Av.Br.Len was not measured in primates, it is relevant to compare their absolute values of Conn.D to those recovered in xenarthrans. In human populations [95], they are much lower (4–5 per mm^3^ against 11.7 per mm^3^ on average in xenarthrans, Table 2). As humans’ ROIs are bigger than those of most xenarthrans, this might be due to scaling (isometrical slope for Conn.D is −3, Mielke et al. (under review); however, this parameter scaled with positive allometry in Doube et al. [8]). The average xenarthran value roughly equals the maximal average value of primates recovered by Shaw and Ryan [98].

Humeral head’s BV/TV was not found as functionally discriminant in xenarthrans. But one can note that the primate values’ range (ca. 0.13–0.41 [93–96, 98]) is lower than the average xenarthran value (0.43). The overall greater bone fraction found in the humeral head of xenarthrans might be related to other factors, as an overarching functional difference between the two clades is not obvious. Straehl et al. [88] analysed the bone histology and structure of mid-diaphyseal sections of the humerus and femur among xenarthrans. As their data, ours indicate that the humeral bone fraction (global compactness was measured therein) does not conspicuously differ among xenarthran clades or functional categories. However, Straehl et al. [88] found that armadillos differ from the other xenarthrans in having a humeral mid-diaphysis that is more compact when compared to that of the femur, and related that to their fossorial habits. It would hence be relevant to compare trabecular architecture in both bones to check if a similar pattern is observed at the epiphyses.

BS/TV was found to be lower in armadillos. One might expect that this feature relates to thicker (or less spaced) trabeculae, but that was not the case in our dataset. It is noteworthy that armadillos’ normalised Tb.Th is conspicuously greater than that of other xenarthrans. So, even though Tb.Th (after size correction) was not found as significantly different among the lifestyle categories, one can assume that there is a trend for armadillos to have thicker trabeculae, involving a lower BS/TV. This in turn can be more easily understood functionally, as armadillos’ forelimbs likely undergo the loads that are the largest in relative magnitude among xenarthrans (maybe to the exception of *Myrmecophaga*). Scherf et al. [94] found average values for the orangutan and humans that are, as expected, lower than in xenarthrans, because the isometric scaling is negative (slope of −1; however, one should note that it scales with slight positive allometry in the femoral head of mammals [8]), but the chimpanzee was characterised by greater values on average. As the latter taxon is assumed to be characterised by the most strenuous lifestyle, it seems inconsistent with our results (and also with Scherf et al.’s [94] expectations).

As in xenarthrans, the individual Tb.Th and Tb.Sp parameters in primates [25, 93] did not yield a clear functional discrimination. In great apes, however, Scherf et al. [94] found that the chimpanzee was characterized by lower Tb.Sp values. In Neolithic humans a tendency to thicker and less spaced trabeculae was pointed out [95]. The higher (normalised) Tb.Th values in armadillos are in accordance with this trend. The primate Tb.Th range ca. 0.14–0.24 mm [93–96] falls below the average xenarthran value (0.26 mm), except for the values of *Homo* and *Pongo* measured by Shaw and Ryan [98], which fall within the xenarthran range (xenarthran max. = 0.45 mm).

### Radius

The radial head was studied by Gebauer et al. [100] in humans (males and females from 20 to 80 years old). In the latter, BV/TV ranges from 0.06 to 0.15, while in xenarthrans it ranges from 0.31 to 0.66 (Additional file 7). Even though the ROI is differently defined therein, it is clear from Gebauer et al.’s [100] figures that xenarthrans’ radial head comprises relatively more bone than that of humans. While more data are necessary to confirm this, it seems consistent to find a much lower bone fraction in the non-weight bearing radial head of humans.

In the radial trochlea, the MDT is found to be mostly proximodistal (therein called superior-inferior) in humans [101]. The DA (therein the eigenvalues ratio is reported) was found to range from 0.33 to 0.45 (mean = 0.41), while in xenarthrans values range from 0.48 to 0.93. As for the comparison of the radial head, it seems that the fact that the distal radius of xenarthrans as a whole is weight bearing is reflected in their clearly greater DA than in humans.

An important limitation of our study is the different resolutions at which the specimens were scanned across our dataset. Even if we followed published recommendation on that regard (involving the relative resolution [57]), one can expect these resolution differences to bias our measurements, especially for those that directly relate to the size of the pixels, namely BS (and its ratio to TV), Tb.Th, Tb.Sp, and Av.Br.Len. This could potentially explain the fact that these parameters were the least discriminant in our analysis. Furthermore, the relevance of Av.Br.Len in functional analyses is not straightforward, as the shape of the trabeculae, which can be from rod-like to plate-like, likely involves conspicuously different mechanical properties ([102] and references therein).

## Conclusions

We present herein a dataset comprised of μCT-scan data characterizing the 3D trabecular architecture of the main forelimb epiphyses of all extant genera of xenarthrans (armadillos, anteaters, and sloths). The important variation observed in most of their trabecular parameters offers a unique insight in the functional relevance of these parameters, as the forelimb of xenarthrans is characterized by conspicuous differences in its functional use. Most parameters did not yield a phylogenetic signal, suggesting that the phylogenetic relationships among xenarthrans are not preponderantly affecting their trabecular architecture. Some trabecular parameters, the degree of anisotropy (DA) in particular, were found to significantly differ among the functional categories, even when body size and phylogeny were taken into account. This suggests that not only the diaphysis, but also the epiphyseal structure of long bones can yield an important functional signal. Indeed, a greater DA seems to be consistently acquired in the epiphyses of the more fossorial xenarthrans. As digging adaptations are widespread among tetrapods, a future endeavour will be to check whether a greater degree of anisotropy also sets other fossorial taxa apart. This could confirm the importance of this parameter for the practice of strenuous activities such as digging. So far, only primates were similarly investigated, so, given the results reported herein, we expect that trabecular architecture will represent a promising research avenue that will be key to reach a better understanding of bone biomechanics in an ecological context as well as for lifestyle/(palaeo)biological reconstructions (applicable both to extinct and extant taxa for which ecological data is lacking).

## Additional files

Additional file 1: Species/specimen list and raw data as measured with BoneJ [61]. Each worksheet corresponds to a region of interest. Note that not all parameters given therein were analysed. Abbreviations: See main text and [103]. (XLSX 94 kb)

Additional file 2: Orientation of the scapula and location of its region of interest (ROI), the glenoid cavity. The 3D pdf includes the superimposed surface models of the whole scapula (by default transparent), ROI (glenoid cavity, orange) and scale (cubic, black). The specimen’s orientation in the coordinate system follows that used in the analyses (the lateral view was set to be by default). The example specimen: *Chlamyphorus truncatus* ZMB_MAM_6007, right scapula. (PDF 8113 kb)

Additional file 3: Orientation of the humerus and location of its regions of interest (ROIs). The 3D pdf includes the superimposed surface models of the whole humerus (by default transparent), ROIs (humeral head and capitulum, orange) and scale (cubic, black). The specimen’s orientation in the coordinate system follows that used in the analyses (the anterior view was set to be by default). The example specimen: *Cabassous tatouay* SMNS-26661, right humerus. (PDF 16352 kb)

Additional file 4: Orientation of the radius and location of its regions of interest (ROIs). The 3D pdf includes the superimposed surface models of the whole radius (by default transparent), ROIs (radial head and trochlea, orange) and scale (cubic, black). The specimen orientation’s in the coordinate system follows that used in the analyses (the anterior view was set to be by default). The example specimen: *Euphractus sexcinctus* SMNS-26660, right radius. (PDF 14211 kb)

Additional file 5: R script to convert eigenvector cosines (as outputted by BoneJ [61]) into azimuth and plunge. Made under R version 3.4.1 [66]. (R 1 kb)

Additional file 6: Distribution of the non-directional trabecular parameters of the regions of interest (ROIs) distal to the glenoid cavity (see Fig. 4 of main text) among the lifestyle categories. Box-plots describing the distribution of the non-directional trabecular parameters of the regions of interest (ROIs) distal to the glenoid cavity (see Fig. 4 of main text) among the lifestyle categories. If the parameter was size-corrected, “sc” precedes its abbreviation, and it is the residuals of the regression of the original parameter against a body size proxy (TV) that are used and plotted (see original parameters’ units in the text). Note that a phylogenetic ANCOVA was warranted in the cases of the scDA for the humeral ROIs (see main text). Abbreviations: arma, armadillos; sloth, sloths; ant, anteaters. Sample size is only given for scDA but is valid for the other parameters as well. (PDF 34 kb)

Additional file 7: Summary of the non-directional trabecular parameters values among xenarthrans for each region of interest. Excel workbook containing three worksheets, for the mean, minimum, and maximum values respectively of the non-directional trabecular parameters among xenarthrans for each region of interest. Abbreviations: Av.Br.Len, average branch length; BS, bone surface; BV, bone volume; Conn.D, connectivity density; DA, degree of anisotropy; Tb.Th, trabecular mean thickness; Tb.Sp, trabecular mean spacing; NU, no units. (XLSX 15 kb)

Additional file 8: *P*-values of the pairwise comparisons among lifestyle categories. Excel workbook containing two worksheets, one for the traditional (non-phylogenetic) comparisons between each pair of category for each ROI and parameter, and the other for the phylogenetically informed comparisons (given only when the latter are warranted). Abbreviations: Av.Br.Len, average branch length; BS, bone surface; BV, bone volume; Conn.D, connectivity density; DA, degree of anisotropy; Tb.Th, trabecular mean thickness; Tb.Sp, trabecular mean spacing; NU, no units. (XLSX 11 kb)

## Trabecular parameters

Av.Br.Len: Average branch length
BS: Bone surface
BV: Bone volume
Conn.D: Connectivity density
DA: Degree of anisotropy
MDT: Main direction of trabeculae
ROI: Region of interest
Tb.Sp: Trabecular mean spacing
Tb.Th: Trabecular mean thickness

## Institutions

CeNaK: Centrum für Naturkunde, Universität Hamburg, Germany
SMF: Naturmuseum Senckenberg, Frankfurt am Main, Germany
SMNS: Staatliches Museum für Naturkunde, Stuttgart, Germany
ZLHUB: Zoologische Lehrsammlung HU-Berlin
ZMB: Museum für Naturkunde Berlin, Germany
ZSM: Zoologische Staatssammlung München, Germany
