# Supplementary material for: Trabecular architecture in the forelimb epiphyses of extant xenarthrans (Mammalia)
Source: Front Zool. 2017 Nov 29;14:52. doi: 10.1186/s12983-017-0241-x (PMC5707916; doi:10.1186/s12983-017-0241-x)
Supplement: Supplementary file 2 — Orientation of the scapula and location of its region of interest (ROI), the glenoid cavity. The 3D pdf includes the superimposed surface models of the whole scapula (by default transparent), ROI (glenoid cavity, orange) and scale (cubic, black). The specimen’s orientation in the coordinate system follows that used in the analyses (the lateral view was set to be by default). The example specimen: Chlamyphorus truncatus ZMB_MAM_6007, right scapula. (PDF 8113 kb) [file 12983_2017_241_MOESM2_ESM.pdf]

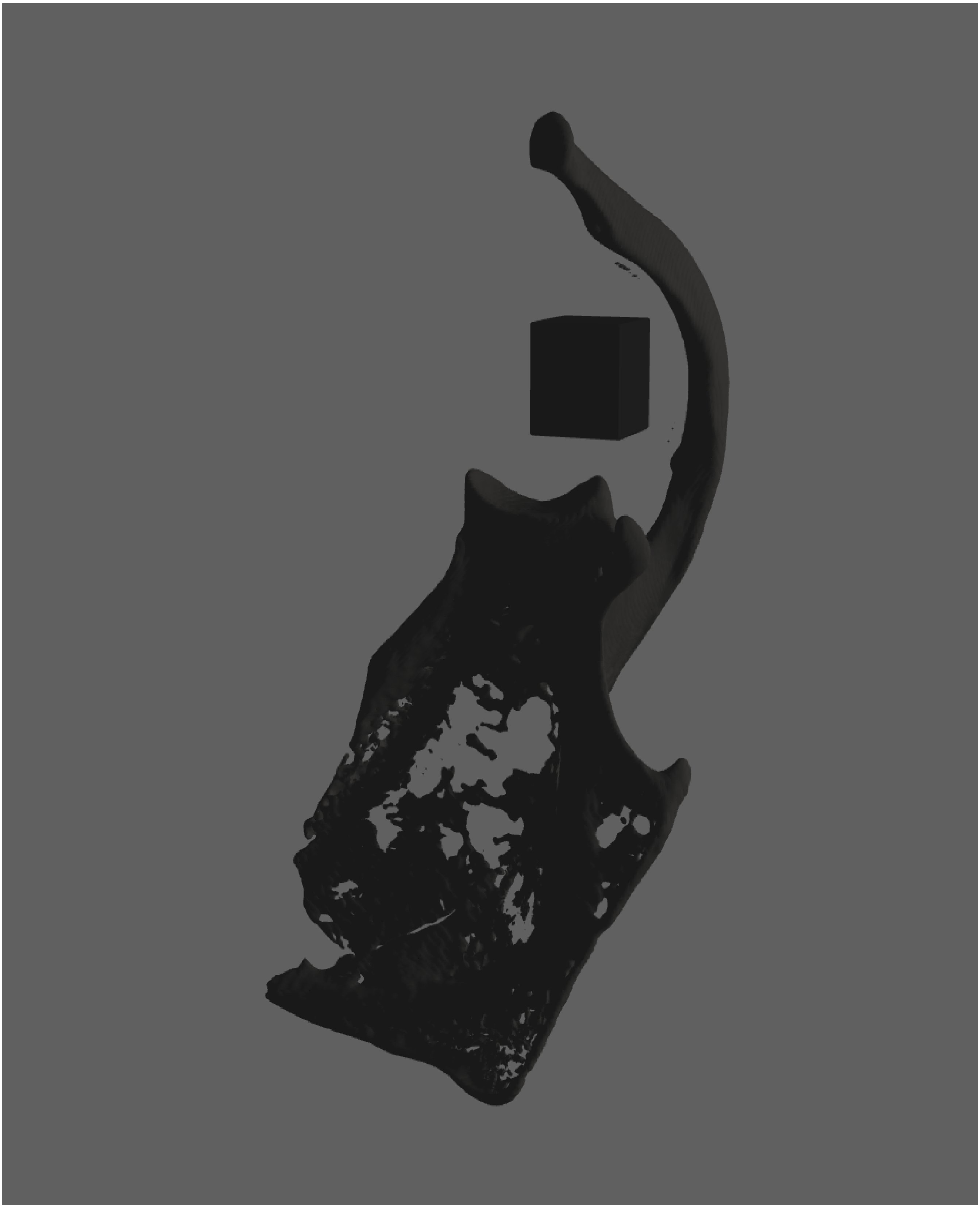

Additional file 2. Orientation of the scapula and location of its region of interest (ROI), the glenoid cavity. The 3D pdf includes the superimposed surface models of the whole scapula (by default transparent), ROI (glenoid cavity, orange) and scale (cubic, black). The specimen's orientation in the coordinate system follows that used in the analyses (the lateral view was set to be by default). The example specimen: *Chlamyphorus truncatus* ZMB_MAM_6007, right scapula.
