# Supplementary material for: Trabecular architecture in the forelimb epiphyses of extant xenarthrans (Mammalia)
Source: Front Zool. 2017 Nov 29;14:52. doi: 10.1186/s12983-017-0241-x (PMC5707916; doi:10.1186/s12983-017-0241-x)
Supplement: Supplementary file 3 — Orientation of the humerus and location of its regions of interest (ROIs). The 3D pdf includes the superimposed surface models of the whole humerus (by default transparent), ROIs (humeral head and capitulum, orange) and scale (cubic, black). The specimen’s orientation in the coordinate system follows that used in the analyses (the anterior view was set to be by default). The example specimen: Cabassous tatouay SMNS-26661, right humerus. (PDF 16352 kb) [file 12983_2017_241_MOESM3_ESM.pdf]

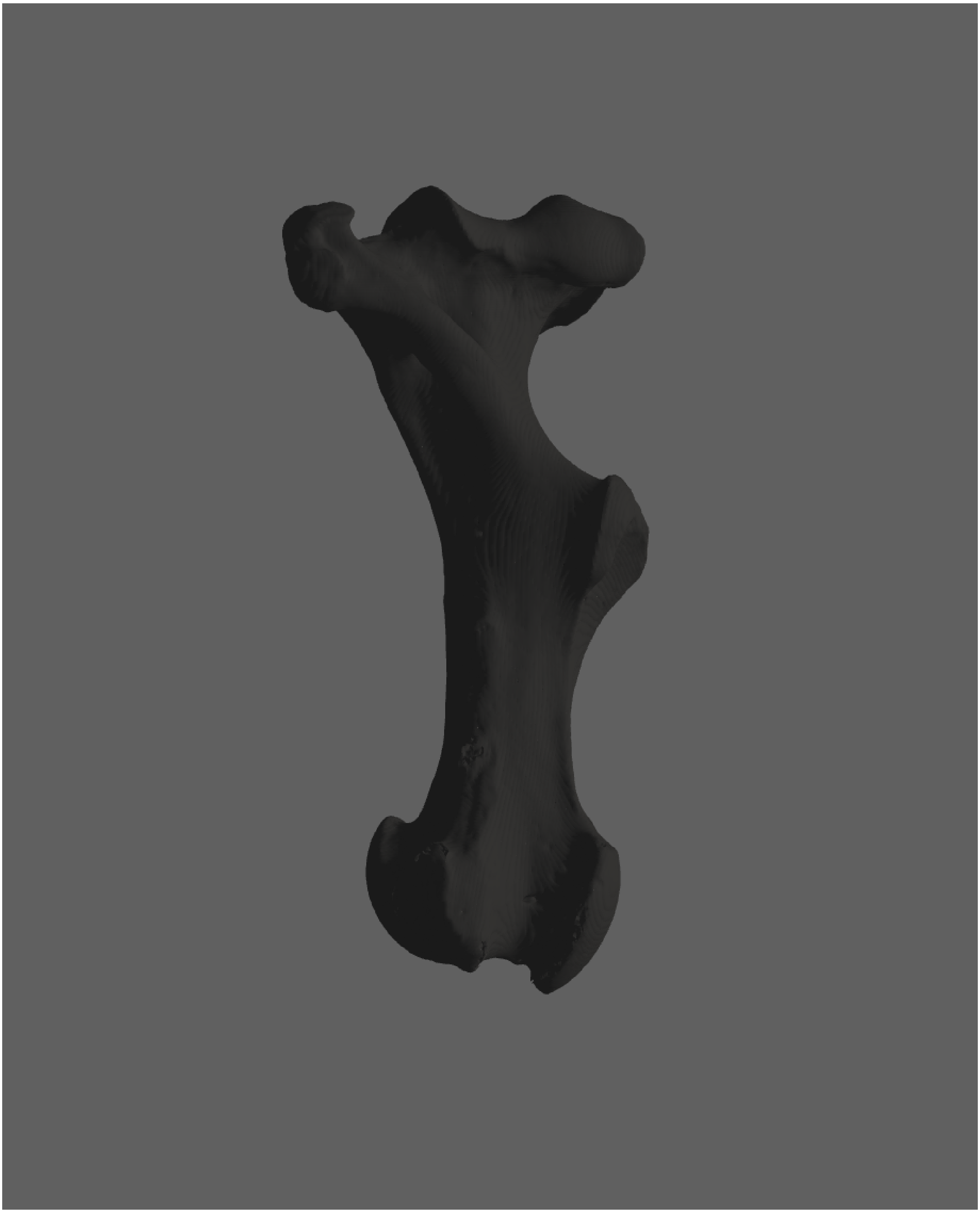

Additional file 3. Orientation of the humerus and location of its regions of interest (ROIs).
The 3D pdf includes the superimposed surface models of the whole humerus (by default transparent), ROIs (humeral head and capitulum, orange) and scale (cubic, black). The specimen's orientation in the coordinate system follows that used in the analyses (the anterior view was set to be by default). The example specimen: *Cabassous tatouay* SMNS-26661, right humerus.
