# Supplementary material for: Trabecular architecture in the forelimb epiphyses of extant xenarthrans (Mammalia)
Source: Front Zool. 2017 Nov 29;14:52. doi: 10.1186/s12983-017-0241-x (PMC5707916; doi:10.1186/s12983-017-0241-x)
Supplement: Supplementary file 4 — Orientation of the radius and location of its regions of interest (ROIs). The 3D pdf includes the superimposed surface models of the whole radius (by default transparent), ROIs (radial head and trochlea, orange) and scale (cubic, black). The specimen orientation’s in the coordinate system follows that used in the analyses (the anterior view was set to be by default). The example specimen: Euphractus sexcinctus SMNS-26660, right radius. (PDF 14211 kb) [file 12983_2017_241_MOESM4_ESM.pdf]

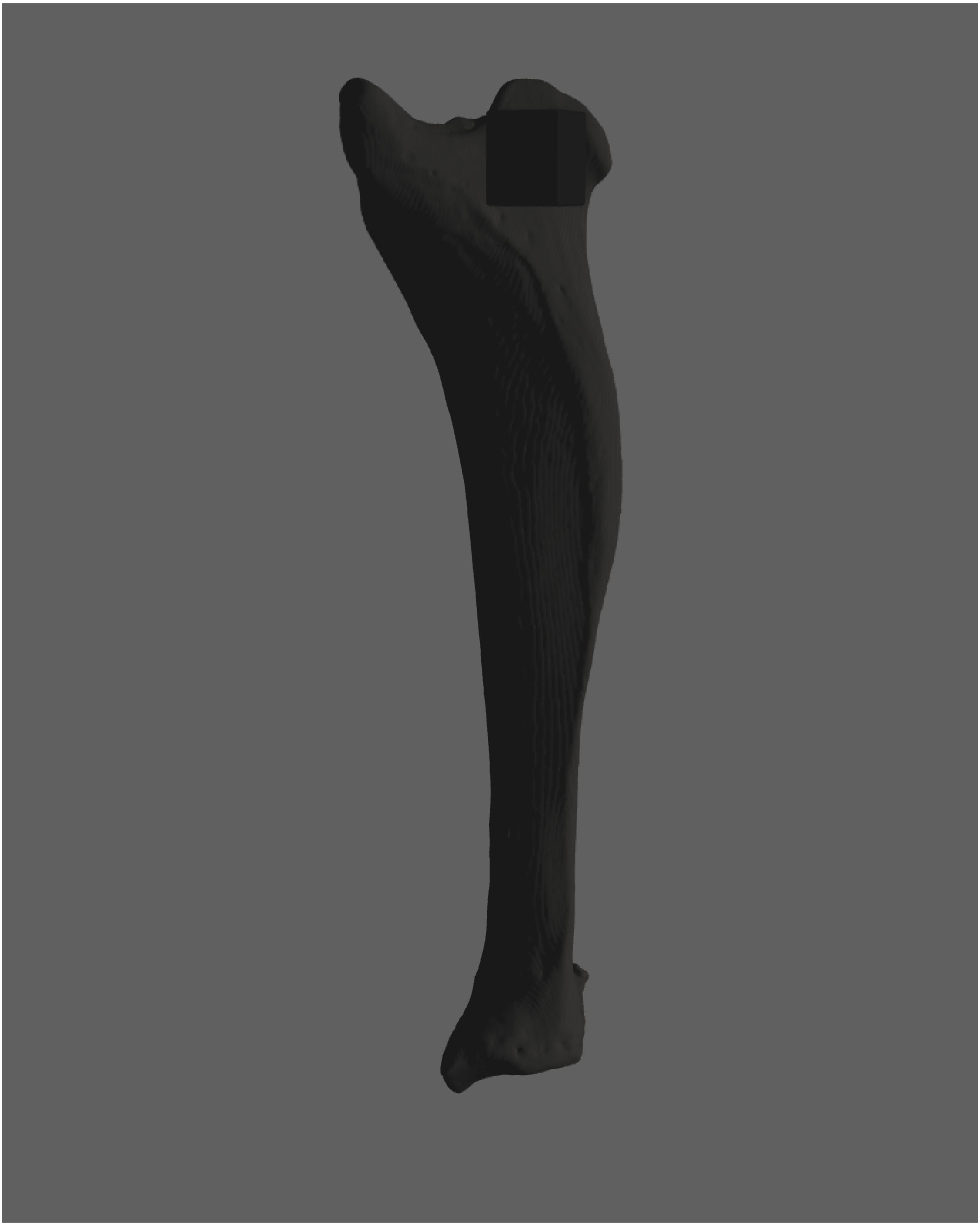

Additional file 4. Orientation of the radius and location of its regions of interest (ROIs).
The 3D pdf includes the superimposed surface models of the whole radius (by default transparent), ROIs (radial head and trochlea, orange) and scale (cubic, black). The specimen orientation's in the coordinate system follows that used in the analyses (the anterior view was set to be by default). The example specimen: *Euphractus sexcinctus* SMNS-26660, right radius.
