# Supplementary material for: Trabecular architecture in the forelimb epiphyses of extant xenarthrans (Mammalia)
Source: Front Zool. 2017 Nov 29;14:52. doi: 10.1186/s12983-017-0241-x (PMC5707916; doi:10.1186/s12983-017-0241-x)
Supplement: Supplementary file 6 — Distribution of the non-directional trabecular parameters of the regions of interest (ROIs) distal to the glenoid cavity (see Fig. 4 of main text) among the lifestyle categories. Box-plots describing the distribution of the non-directional trabecular parameters of the regions of interest (ROIs) distal to the glenoid cavity (see Fig. 4 of main text) among the lifestyle categories. If the parameter was size-corrected, “sc” precedes its abbreviation, and it is the residuals of the regression of the original parameter against a body size proxy (TV) that are used and plotted (see original parameters’ units in the text). Note that a phylogenetic ANCOVA was warranted in the cases of the scDA for the humeral ROIs (see main text). Abbreviations: arma, armadillos; sloth, sloths; ant, anteaters. Sample size is only given for scDA but is valid for the other parameters as well. (PDF 34 kb) [file 12983_2017_241_MOESM6_ESM.pdf]

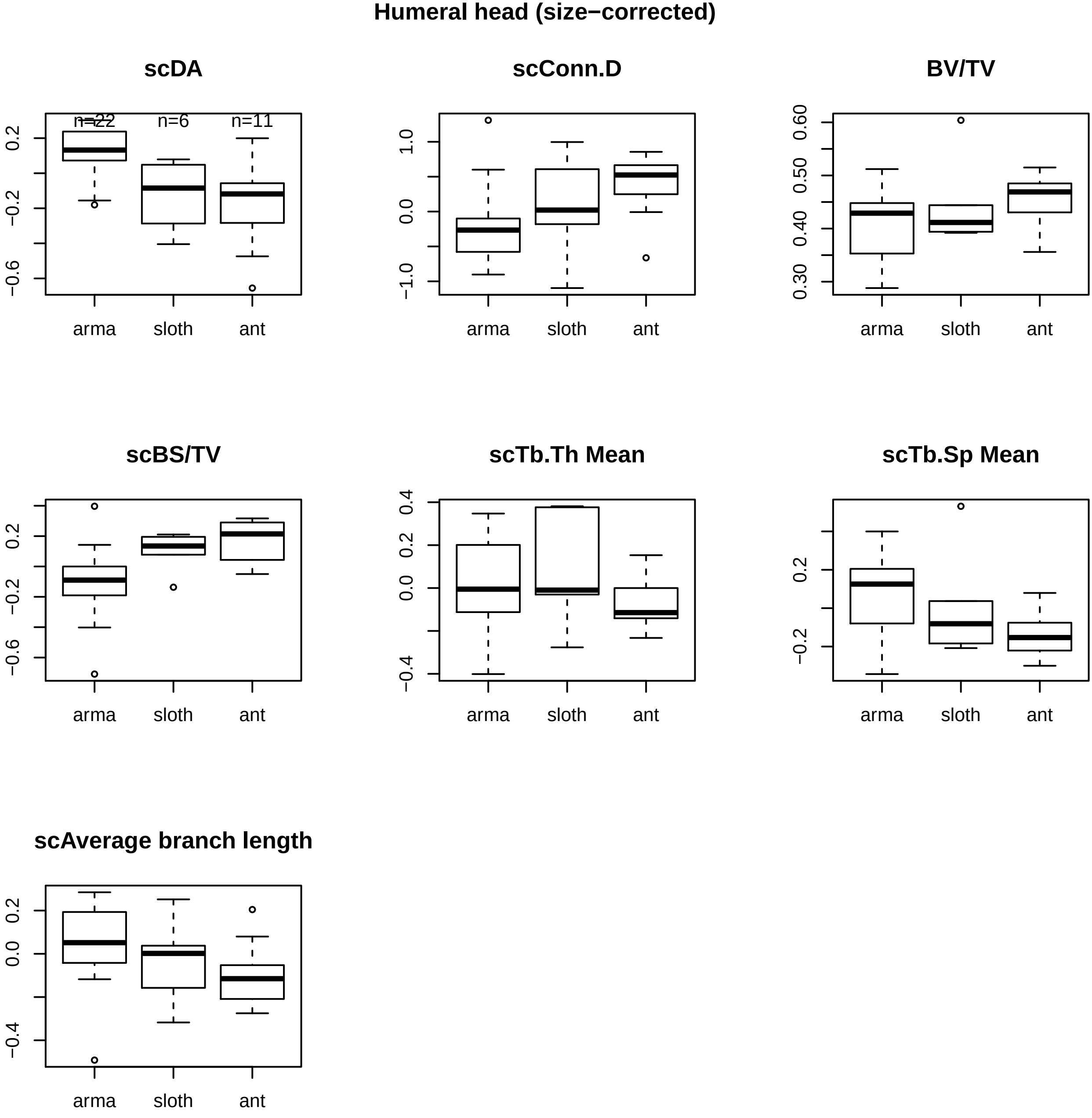
Humeral head (size−corrected)
scDA
n=22
n=6
n=11
scConn.D
BV/TV
scBS/TV
scTb.Th Mean
scTb.Sp Mean
scAverage branch length
arma
sloth
ant

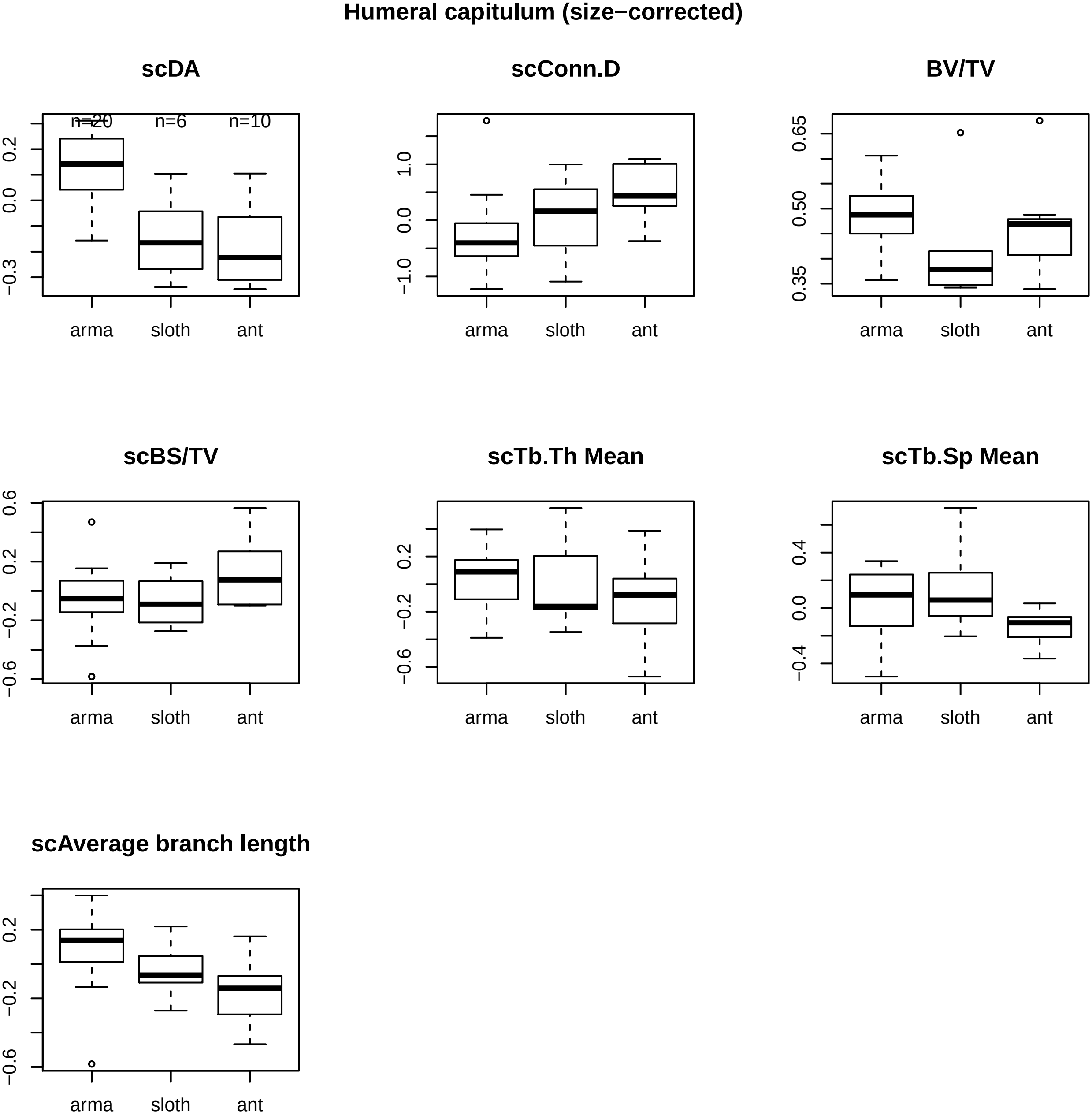
Humeral capitulum (size−corrected)
scDA
n=20
n=6
n=10
−0.3
0.0
0.2
scConn.D
−1.0
0.0
1.0
BV/TV
0.35
0.50
0.65
scBS/TV
−0.6
−0.2
0.2
0.6
scTb.Th Mean
−0.6
−0.2
0.2
scTb.Sp Mean
−0.4
0.0
0.4
scAverage branch length
−0.6
−0.2
0.2
arma
sloth
ant

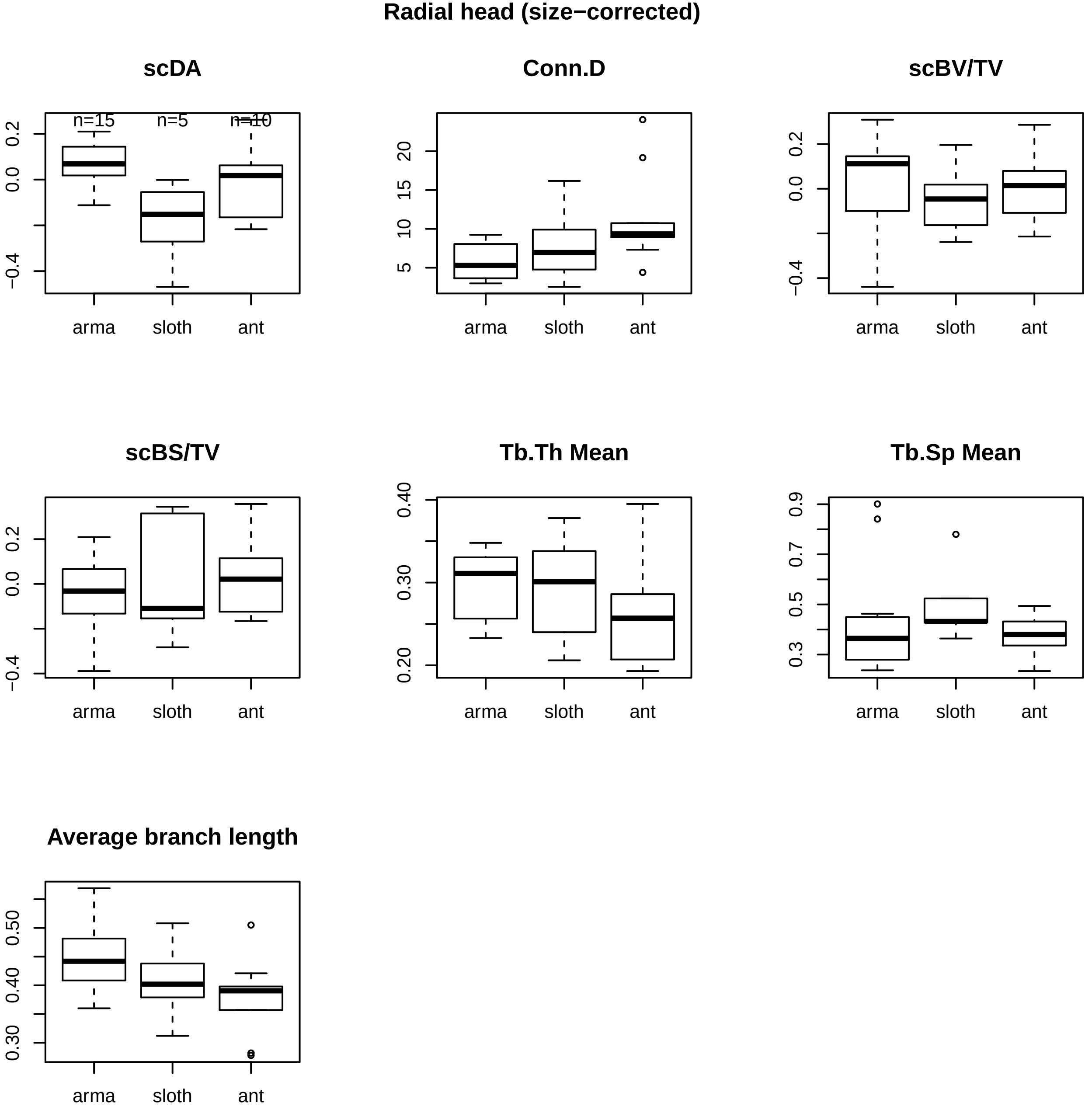

Radial head (size−corrected)
scDA
n=15
n=5
n=10
0.2
0.0
−0.4
arma
sloth
ant
Conn.D
5
10
15
20
arma
sloth
ant
scBV/TV
0.2
0.0
−0.4
arma
sloth
ant
scBS/TV
0.2
0.0
−0.4
arma
sloth
ant
Tb.Th Mean
0.20
0.30
0.40
arma
sloth
ant
Tb.Sp Mean
0.3
0.5
0.7
0.9
arma
sloth
ant
Average branch length
0.30
0.40
0.50
arma
sloth
ant

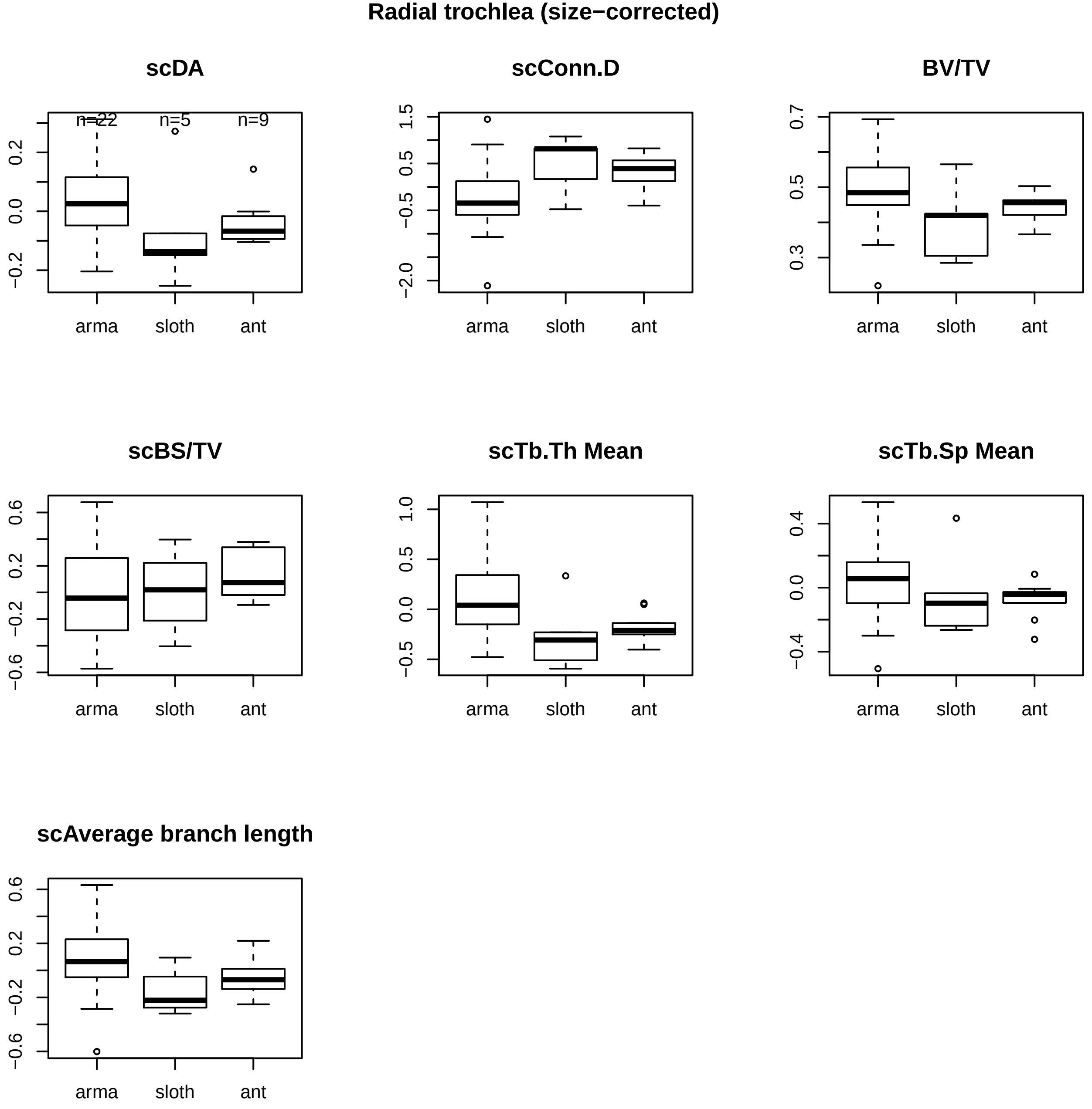
Radial trochlea (size−corrected)
scDA
n=22
n=5
n=9
0.2
0.0
−0.2
arma
sloth
ant
scConn.D
1.5
0.5
−0.5
−2.0
arma
sloth
ant
BV/TV
0.7
0.5
0.3
arma
sloth
ant
scBS/TV
0.6
0.2
−0.2
−0.6
arma
sloth
ant
scTb.Th Mean
1.0
0.5
0.0
−0.5
arma
sloth
ant
scTb.Sp Mean
0.4
0.0
−0.4
arma
sloth
ant
scAverage branch length
0.6
0.2
−0.2
−0.6
arma
sloth
ant
